# P067 Diagnostic and treatment dilemmas in atypical giant cell arteritis

**DOI:** 10.1093/rap/rkag113.097

**Published:** 2026-11-03

**Authors:** Hassan Hussain, Claire MacFadyen, Ritu Malaiya

**Affiliations:** St Helier’s Hospital, London, United Kingdom; St Helier’s Hospital, London, United Kingdom; St Helier’s Hospital, London, United Kingdom

## Abstract

**Introduction:**

Our patient is a 77-year-old lady who struggled with steroid-responsive atypical headaches over the course of a decade. She had a history of episcleritis and uveitis and was a lifelong smoker with hypercholesterolemia.

Multiple investigations, including two temporal artery biopsies, were not conclusive. A diagnosis of giant cell arteritis with aortitis was finally unequivocally confirmed in 2025 following a PET CT. Her management has been complicated by numerous debilitating side effects to both standard steroid therapy and steroid sparing agents, culminating in an episode of bowel perforation following tocilizumab therapy.

**Case description:**

Our patient’s symptoms initially developed in 2013 with a headache and fever. A steroid course prescribed by her GP for a concurrent chest infection temporarily resolved her symptoms. In January 2014 she presented to the Acute Medical Unit with sudden onset reduced visual acuity in her right eye and a headache on the top of her head. Examination was unremarkable, with no ophthalmic GCA identified. Inflammatory markers were mildly elevated. She was started on prednisolone and referred to rheumatology. Subsequent temporal artery biopsy, performed within 2 weeks, was negative. Steroid was eventually weaned, and she was discharged with no ongoing symptoms. 9 years later, in 2023 she represented with 6 months of an occipital headache, left sided temporal tenderness, and blurred vision in both eyes but no ophthalmic GCA was identified. A temporal artery ultrasound was borderline, and she was restarted on prednisolone. A second temporal artery biopsy, performed within 2 weeks, was again negative. Her symptoms resolved with steroid therapy, with the unfortunate consequence of a plethora of steroidal side effects. Attempts to wean her steroids led to a flare of her symptoms, and methotrexate was introduced as a steroid sparing agent. Unfortunately, methotrexate had to be suspended 3 months later due to drug-induced transaminitis. Prednisolone was eventually weaned and discontinued. She remained symptom free until December 2025 when she re-presented systemically unwell with weight loss, fevers, and proximal myalgia. A PET CT identified diffuse aortitis, confirming a diagnosis of large vessel vasculitis/GCA. Steroid was re-commenced alongside tocilizumab. Shortly after starting tocilizumab in January 2026, she was admitted with diverticulitis and localised bowel perforation which was managed conservatively. As a result, further tocilizumab or upadacitinib are now contraindicated, and she remains on a weaning course of steroids with persistent side effects.

**Discussion:**

Throughout our patient’s clinical course, diagnosis remained uncertain. Her headaches were not classical for GCA. Inflammatory markers were not persistently elevated and often did not correspond to the severity of her symptoms. Although 2 ultrasounds showed evidence of possible arteritis, both of her biopsies showed no suggestion of arteritis or disruption of the elastic lamina. It took a PET CT to finally equivocally confirm her disease and allow her to qualify for advanced therapies.

Treatments were hampered by multiple side effects. Standard first line therapy with steroids has been long known to induce multiple metabolic and mood side effects. Our patient suffered with the full spectrum of steroid-induced side effects even at the lowest possible dose to control her symptoms, including mood changes, insomnia, skin fragility, cataracts, diabetes, hypertension, and osteoporosis with a fragility fracture. These side effects understandably lead to an intolerable burden on her quality of life. Subsequent trials with both methotrexate and tocilizumab led to clinically adverse outcomes resulting in both being contraindicated for continued use. Our patient remains on a weaning course of steroids, and her case highlights the need for further steroid sparing agents in the treatment of GCA.

**Key learning points:**

• This case demonstrates the difficulty of certainty in clinical diagnoses where there are no specific biomarkers of disease. Clinicians will often have to rely on their history taking skills: the symptom of a steroid responsive headache throughout the disease course was a clue to the inflammatory nature of the patient’s disease. Imaging also plays a useful tool in modern clinical practice, with two temporal artery US and a PET CT demonstrating evidence of arterial inflammation.

• Our patient was counselled on the importance of sick day rules when taking her tocilizumab–however during and after her bowel perforation she continued to take this medicine. Fortunately, she was seen within one week of discharge and remained clinically well with a full recovery following the diverticular perforation.

• Along with the well documented side effects of steroid treatment, our patient also suffered from bowel perforation secondary to diverticulitis and likely exacerbated by her recently started tocilizumab. This is a documented but rare side effect of tocilizumab but is not specifically listed as such on the Arthritis UK (formerly Versus Arthritis) patient information leaflet. Clinicians should remember to incorporate rarer side effects when choosing and consenting a new treatment and reviewing acutely unwell patients on the wards.

